# Current progress on bio-based polymers and their future trends

**DOI:** 10.1186/2194-0517-2-8

**Published:** 2013-03-18

**Authors:** Ramesh P Babu, Kevin O'Connor, Ramakrishna Seeram

**Affiliations:** 1 Centre for Research Adoptive Nanostructures and Nano Devices, Trinity College, Dublin 2, Ireland; 2 School of Physics, Trinity College Dublin, Dublin 2, Ireland; 3 School of Biomolecular and Biomedical Sciences, Centre for Synthesis and Chemical Biology, UCD Conway Institute, and Earth Institute, University College Dublin, Belfield, Dublin 4, Ireland; 4 NUSNNI, National University of Singapore, 2 Engineering Drive 3, Singapore, 117581 Singapore; 5 Institute of Materials Research and Engineering, Singapore, 117602 Singapore; 6 Jinan University, Guangzhou, China

**Keywords:** Bio-based polymers, Renewable resources, Biotechnologies, Sustainable materials

## Abstract

This article reviews the recent trends, developments, and future applications of bio-based polymers produced from renewable resources. Bio-based polymers are attracting increased attention due to environmental concerns and the realization that global petroleum resources are finite. Bio-based polymers not only replace existing polymers in a number of applications but also provide new combinations of properties for new applications. A range of bio-based polymers are presented in this review, focusing on general methods of production, properties, and commercial applications. The review examines the technological and future challenges discussed in bringing these materials to a wide range of applications, together with potential solutions, as well as discusses the major industry players who are bringing these materials to the market.

**Electronic supplementary material:**

The online version of this article (doi:10.1186/2194-0517-2-8) contains supplementary material, which is available to authorized users.

## Review

### Introduction

Bio-based polymers are materials which are produced from renewable resources. The terms bio-based polymers and biodegradable polymers are used extensively in the literature, but there is a key difference between the two types of polymers. Biodegradable polymers are defined as materials whose physical and chemical properties undergo deterioration and completely degrade when exposed to microorganisms, carbon dioxide (aerobic) processes, methane (anaerobic processes), and water (aerobic and anaerobic processes). Bio-based polymers can be biodegradable (e.g., polylactic acid) or nondegradable (e.g., biopolyhethylene). Similarly, while many bio-based polymers are biodegradable (e.g., starch and polyhydroxyalkanoates), not all biodegradable polymers are bio-based (e.g., polycaprolactone).

Bio-based polymers still hold a tiny fraction of the total global plastic market. Currently, biopolymers share less than 1% of the total market. At the current growth rate, it is expected that biopolymers will account for just over 1% of polymers by 2015 (Doug 2010).

**Figure 1:**
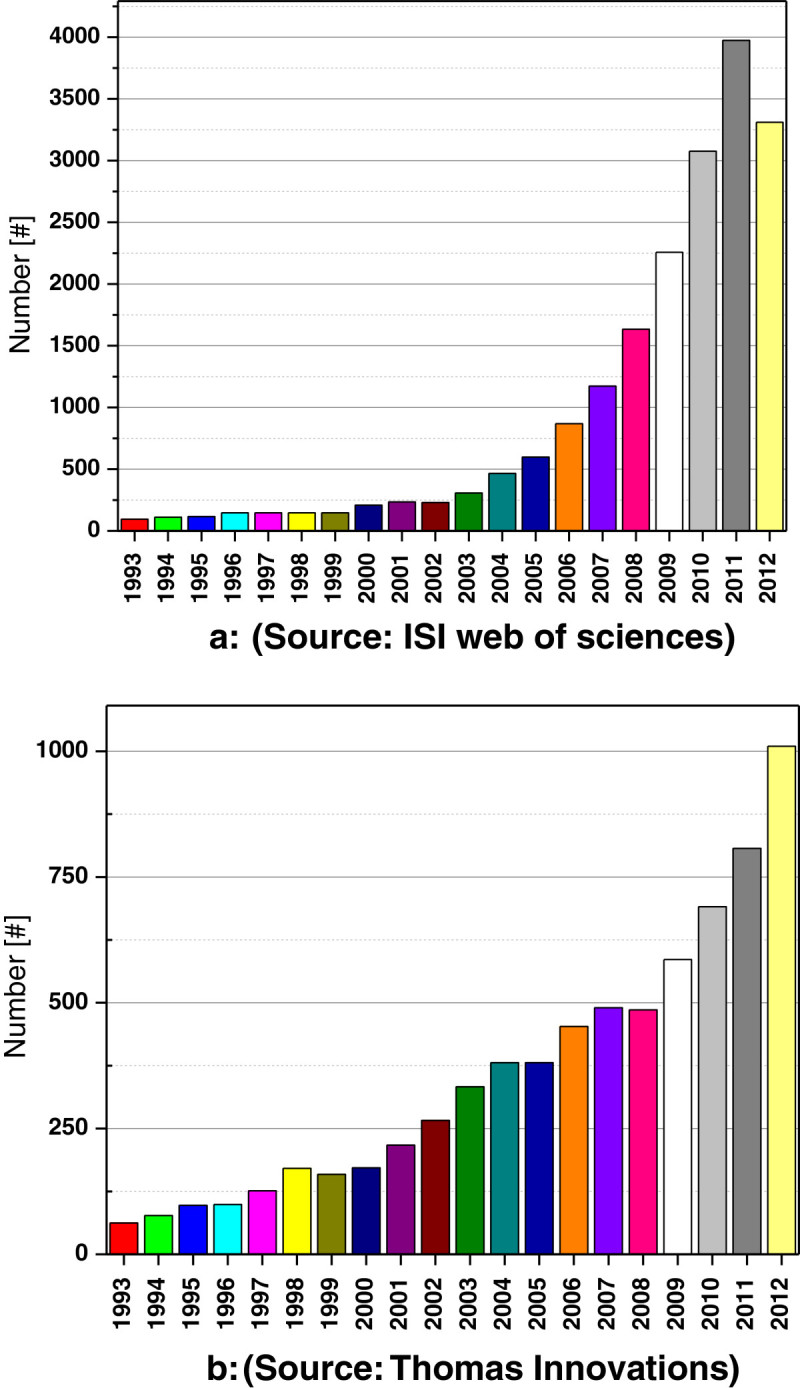
**Citation trends of (a) publications and (b) patents on bio-based polymers in recent years.**

The worldwide interest in bio-based polymers has accelerated in recent years due to the desire and need to find non-fossil fuel-based polymers. As indicated by ISI Web of Sciences and Thomas Innovations, there is a tremendous increase in the number of publication citations on bio-based polymers and applications in recent years, as shown in Figure 1 (Chen and Martin 2012).

Bio-based polymers offer important contributions by reducing the dependence on fossil fuels and through the related positive environmental impacts such as reduced carbon dioxide emissions. The legislative landscape is also changing where bio-based products are being favored through initiatives such as the *Lead Market Initiative* (European Union) and *BioPreferred* (USA). As a result, there is a worldwide demand for replacing petroleum-derived raw materials with renewable resource-based raw materials for the production of polymers.

The first generation of bio-based polymers focused on deriving polymers from agricultural feedstocks such as corn, potatoes, and other carbohydrate feedstocks. However, the focus has shifted in recent years due to a desire to move away from food-based resources and significant breakthroughs in biotechnology. Bio-based polymers similar to conventional polymers are produced by bacterial fermentation processes by synthesizing the building blocks (monomers) from renewable resources, including lignocellulosic biomass (starch and cellulose), fatty acids, and organic waste. Natural bio-based polymers are the other class of bio-based polymers which are found naturally, such as proteins, nucleic acids, and polysaccharides (collagen, chitosan, etc.). These bio-based polymers have shown enormous growth in recent years in terms of technological developments and their commercial applications. There are three principal ways to produce bio-based polymers using renewable resources:

1. Using natural bio-based polymers with partial modification to meet the requirements (e.g., starch)
2. Producing bio-based monomers by fermentation/conventional chemistry followed by polymerization (e.g., polylactic acid, polybutylene succinate, and polyethylene)
3. Producing bio-based polymers directly by bacteria (e.g., polyhydroxyalkanoates).

**Figure 2:**
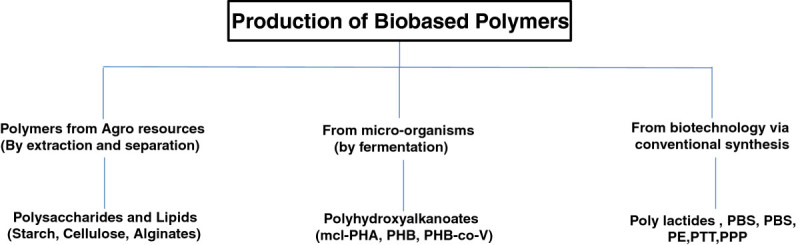
**Most common categories of bio-based polymers produced by various processes.** From Luc and Eric (2012).

In this paper, an overview of bio-based polymers made from renewable resources and natural polymers derived from plant and animal origins is presented. The review will focus on the preparation, properties, applications, and future trends for bio-based polymers. This paper discusses the use of renewable resources such as lignocellulosic biomass to create monomers and polymers that can replace petroleum-based polymers, such as polyester, polylactic acids, and other natural bio-based polymers, which are presented in Figure 2.

### Polylactic acid

Polylactic acid (PLA) has been known since 1845 but not commercialized until early 1990. PLA belongs to the family of aliphatic polyesters with the basic constitutional unit lactic acid. The monomer lactic acid is the hydroxyl carboxylic acid which can be obtained via bacterial fermentation from corn (starch) or sugars obtained from renewable resources. Although other renewable resources can be used, corn has the advantage of providing a high-quality feedstock for fermentation which results in a high-purity lactic acid, which is required for an efficient synthetic process. l-lactic acid or d-lactic acid is obtained depending on the microbial strain used during the fermentation process.

PLA can be synthesized from lactic acid by direct polycondensation reaction or ring-opening polymerization of lactide monomer. However, it is difficult to obtain high molecular weight PLA via polycondensation reaction because of water formation during the reaction. Nature Works LLC (previously Cargill Dow LLC) has developed a low-cost continuous process for the production of PLA (Erwin et al. 2007). In this process, low molecular weight pre-polymer lactide dimers are formed during a condensation process. In the second step, the pre-polymers are converted into high molecular weight PLA via ring-opening polymerization with selected catalysts. Depending on the ratio and stereochemical nature of the monomer (l or d), various types of PLA and PLA copolymers can be obtained. The final properties of PLA produced are highly dependent on the ratio of the d and l forms of the lactic acid which are listed in Table 1 for various blend ratios (Garlotta 2001).

**Table 1:** **Variation in glass transition and melting temperature of PLA with various ratios of L-monomer composition**

Copolymer ratio	Glass transition ( ***T*** g),°C	Melting temperature ( ***T*** m),°C
100:0 (l/dl)-PLA	63	178
95:5 (l/dl)-PLA	59	164
90:10 (l/dl)-PLA	56	150
85:15 (l/dl)-PLA	56	140
80:20 (l/dl)-PLA	56	125

PLA is a commercially interesting polymer as it shares some similarities with hydrocarbon polymers such as polyethylene terephthalate (PET). It has many unique characteristics, including good transparency, glossy appearance, high rigidity, and ability to tolerate various types of processing conditions.

PLA is a thermoplastic polymer which has the potential to replace traditional polymers such as PET, PS, and PC for packaging to electronic and automotive applications (Majid et al. 2010). While PLA has similar mechanical properties to traditional polymers, the thermal properties are not attractive due to low *T* g of 60°C. This problem can be overcome by changing the stereochemistry of the polymer and blending with other polymers and processing aids to improve the mechanical properties, e.g., varying the ratio of l and d isomer ratio strongly influences the crystallinity of the final polymer. However, much more work is required to improve the properties of PLA to suit various applications.

Currently, Nature Works LLC, USA, is the major supplier of PLA sold under the brand name Ingeo, with a production capacity of 100,000 ton/year. There are other manufactures of PLA based in the USA, Europe, China, and Japan developing various grades of PLA suitable for different industrial sectors such as automobile, electronics, medical devices, and commodity applications, which are mentioned in Table 2) (Doug 2010; Ravenstijn 2010).

**Table 2:** **Global suppliers of PLA**

Company	Location	Brand name	Production/planned capacity
			(kton/year)
Nature Works	USA	Ingeo	140 (by 2013)
Futerro	Belgium	Futerro	1.5 (by 2010)
Tate & Lyle	Netherlands	Hycail	0.2 (by 2012)
Purac	Netherlands	Purasorb	0.05
Hiusan Biosciences	China	Hisun	5
Jiangsu Jiulding	China		5
Teijin	Japan	Biofront	1
Toyobo	Japan	Vylocol	0.2
Synbra	Netherlands	Biofoam	50

PLA is widely used in many day-to-day applications. It has been mainly used in food packing (including food trays, tableware such as plates and cutlery, water bottles, candy wraps, cups, etc.). Although PLA has one of the highest heat resistances and mechanical strengths of all bio-based polymers, it is still not suitable for use in electronic devices and other engineering applications. NEC Corporation (Japan) recently produced a PLA with carbon and kenaf fibers with improved thermal and flame retardancy properties. Fujitsu (Japan) developed a polycarbonate blend with PLA to make computer housings. In recent years, PLA has been employed as a membrane material for use in automotive and chemical industry.

The ease of melt processing has led to the production of PLA fibers, which are increasingly accepted in a wide variety of textiles from dresses to sportswear, furnishing to drapes, and soft nonwoven baby wipes to tough landscape textiles. These textiles can outperform traditional textiles made from synthetic counterparts. Bioresorbable scaffolds produced with PLA and various PLA blends are used in implants for growing living cells. The US Food and Drug Administration (FDA) has approved the use of PLA for certain human clinical applications (Dorozhkin 2009; Garlotta 2001). In addition, PLA-based materials have been used for bone support splints. Applications of PLA-based polymers in various fields are listed in Table 3.

**Table 3:** **Application of PLA and their blends in various fields**

Polymer	Applications	Reference
PLGA/PGA	Ovine pulmonary valve replacement	Williams et al. 1999; Sodian et al. 1999, 2000; Cheng et al. 2009
PLA/chitosan PLA/PLGA/chitosan PLA	Drug carrier/drug release	Jeevitha and Kanchana 2013; Jayanth and Vinod 2012; Nagarwal et al. 2010; Chandy et al. 2000; Valantin et al. 2003
PLGA and copolymers	Degradable sutures	Rajev 2000
PLA/HA composites	Porous scaffolds for cellular applications	Jung-Ju et al. 2012
PLA-CaP and PLGA-CaP	Bone fixation devices, plates, pins, screws, and wires, orthopedic applications	Huan et al. 2012
PDLLA	Coatings on metal implants	Schmidmaier et al. 2001
PLA/PLGA	Use in cell-based gene therapy for cardiovascular diseases, muscle tissues, bone and cartilage regeneration, and other treatments of cardiovascular and neurological conditions	Coutu et al. 2009; Kellomaki et al. 2000; Papenburg et al. 2009
PLA and PLA blends	Packaging films, commodity containers, electrical appliances, mobile phone housings, floor mats, automotive spare parts	Rafael et al. 2010
PLA	Textile applications	Gupta et al. 2007; Avinc and Akbar 2009

PLGA, polylactic acid-*co*-glycolic acid; CaP, calcium phosphates; HA, hydroxyapatite.

### Polyhydroxyalkanoates

Polyhydroxyalkanoates (PHAs) are a family of polyesters produced by bacterial fermentation with the potential to replace conventional hydrocarbon-based polymers. PHAs occur naturally in a variety of organisms, but microorganisms can be employed to tailor their production in cells. Polyhydroxybutyrate (PHB), the simplest PHA, was discovered in 1926 by Maurice Lemoigne as a constituent of the bacterium *Bacillus megaterium* (Lemoigne 1923).

PHA can be produced by varieties of bacteria using several renewable waste feedstocks. A generic process to produce PHA by bacterial fermentation involves fermentation, isolation, and purification from fermentation broth. A large fermentation vessel is filled with mineral medium and inoculated with a seed culture that contains bacteria. The feedstocks include cellulosics, vegetable oils, organic waste, municipal solid waste, and fatty acids depending on the specific PHA required. The carbon source is fed into the vessel until it is consumed and cell growth and PHA accumulation is complete. In general, a minimum of 48 h is required for fermentation time. To isolate and purify PHA, cells are concentrated, dried, and extracted with solvents such as acetone or chloroform. The residual cell debris is removed from the solvent containing dissolved PHA by solid-liquid separation process. The PHA is then precipitated by the addition of an alcohol (e.g., methanol) and recovered by a precipitation process (Kathiraser et al. 2007).

More than 150 PHA monomers have been identified as the constituents of PHAs (Steinbüchel and Valentin 1995). Such diversity allows the production of bio-based polymers with a wide range of properties, tailored for specific applications. Poly-3-hydroxybutyrate was the first bacterial PHA identified. It has received the greatest attention in terms of pathway characterization and industrial-scale production. It possesses similar thermal and mechanical properties to those of polystyrene and polypropylene (Savenkova et al. 2000). However, due to its slow crystallization, narrow processing temperature range, and tendency to ‘creep’, it is not attractive for many applications, requiring development in order to overcome these shortcomings (Reis et al. 2008). Several companies have developed PHA copolymers with typically 80% to 95% (*R*)-3-hydroxybutyric acid monomer and 5% to 20% of a second monomer in order to improve the properties of PHAs. Some specific examples of PHAs include the following:

- Poly(3HB): Poly(3-hydroxybutyrate)
- Poly(3HB-*co*-3HV): Poly(3-hydroxybutyrate-*co*-3-hydroxyvalerate), PHBV
- Poly(3-HB-*co*-4HB): Poly(3-hydroxybutyrate-*co*-4-hydroxybutyrate)
- Poly(3HB-*co*-3HH): Poly(3-hydroxyoctanoate-*co*-hydroxyhexanoate)
- Poly(3HO-*co*-3HH): Poly(3-hydroxyoctanoate-*co*-hydroxyhexanoate)
- Poly (4-HB): Poly(4-hydroxybutyrate).

The copolymer poly(3HB-*co*-3HV) has a much lower crystallinity, decreased stiffness and brittleness, and increased tensile strength and toughness compared to poly(3HB) while remaining biodegradable. It also has a higher melt viscosity, which is a desirable property for extrusion and blow molding (Hanggi 1995).

The first commercial plant for PHBV was built in the USA in a joint venture between Metabolix and Archer Daniels Midland. However, the joint venture between these two companies ended in 2012. Currently, Tianan Biologic Material Co. in China is the largest producer of PHB and PHB copolymers. Tianan's PHBV contains about 5% valerate which improves the flexibility of the polymer. Tainjin Green Biosciences, China, invested along with DSM to build a production plant with 10-kton/year capacity to produce PHAs for packing and biomedical applications (DSM press release 2008). The current global manufacturers of PHB-based polymers are listed in Table 4 (Doug 2010; Ravenstijn 2010).

**Table 4:** **Global suppliers of various types of PHAs**

Company	Location	Brand name	Production/planned capacity (kton/year)
Bio-on	Italy	Minerv	10
Kaneka	Singapore		10 (by 2013)
Meredian	USA		13.5
Metabolix	USA	Mirel	50
Mitsubishi Gas Chemicals	Japan	Biogreen	0.05
PHB Industrial S/A	Brazil	Biocycle	0.05
Shenzen O'Bioer	China		
TEPHA	USA	ThephaFLEX/ThephELAST	
Tianan Biological Materials	China	Enmat	2
Tianjin Green Biosciences	China	Green Bio	10
Tianjin Northern Food	China		
Yikeman Shandong	China		3

PHA polymers are thermoplastic, and their thermal and mechanical properties depend on their composition. The *T* g of the polymers varies from −40°C to 5°C, and the melting temperatures range from 50°C to 180°C, depending on their chemical composition (McChalicher and Srienc 2007). PHB is similar in its material properties to polypropylene, with a good resistance to moisture and aroma barrier properties. Polyhydroxybutyric acid synthesized from pure PHB is relatively brittle and stiff. PHB copolymers, which may include other fatty acids such as beta-hydroxyvaleric acid, may be elastic (McChalicher and Srienc 2007).

PHAs can be processed in existing polymer-processing equipment and can be converted into injection-molded components: film and sheet, fibers, laminates, and coated articles; nonwoven fabrics, synthetic paper products, disposable items, feminine hygiene products, adhesives, waxes, paints, binders, and foams. Metabolix has received FDA clearance for use of PHAs in food contact applications. These materials are suitable for a wide range of food packing applications including caps and closures, disposable items such as forks, spoons, knives, tubs, trays, and hot cup lids, and products such as housewares, cosmetics, and medical packaging (Philip et al. 2007).

PHA and its copolymers are widely used as biomedical implant materials. Various applications of PHA and their polymer blends are listed in Table 5. These include sutures, suture fasteners, meniscus repair devices, rivets, bone plates, surgical mesh, repair patches, cardiovascular patches, tissue repair patches, and stem cell growth. Changing the PHA composition allows the manufacturer to tune the properties such as biocompatibility and polymer degradation time within desirable time frames under specific conditions. PHAs can also be used in drug delivery due to their biocompatibility and controlled degradability. Only a few examples of PHAs have been evaluated for this type of applications, and it remains an important area for exploitation (Tang et al. 2008).

**Table 5:** **Application of PHAs and their blends in various fields**

PHA polymer type	Applications	Reference
P(3HB), P(3HB-*co*-3HHX) and blends	Scaffolds, nerve regeneration, soft tissue, artificial esophagus, drug delivery, skin regeneration, food additive	Yang et al. 2002; Chen and Qiong 2005; Bayram and Denbas 2008; Tang et al. 2008; Clarinval and Halleux 2005
mcl-PHA/scl-PHA	Cardiac tissue engineering, drug delivery, cosmetics, drug molecules	Sodian et al. 2000; Wang et al. 2003; de Roo et al. 2002; Zhao et al. 2003; Ruth et al. 2007
P(4HB) and P(3HO)	Heart valve scaffolds, food additive	Clarinval and Halleux 2005; Valappil et al. 2006
P(3HB-*co*-4HB), P(3HB-*co*-3HV)	Drug delivery, scaffolds, artificial heart values, patches to repair gastrointestinal tracts, sutures	Türesin et al. 2001; Williams et al. 1999; Chen et al. 2008; Freier et al. 2002; Kunze et al. 2006; Volova et al. 2003
PHB, Mirel P103	Commodity applications, shampoo and cosmetic bottles, cups and food containers	Philip et al. 2007; Amass et al. 1998; Walle et al. 2001

### Polybutylene succinate

Polybutylene succinate (PBS) is an aliphatic polyester with similar properties to those of PET. PBS is produced by condensation of succinic acid and 1,4-butanediol. PBS can be produced by either monomers derived from petroleum-based systems or the bacterial fermentation route. There are several processes for producing succinic acid from fossil fuels. Among them, electrochemical synthesis is a common process with high yield and low cost. However, the fermentation production of succinic acid has numerous advantages compared to the chemical process. Fermentation process uses renewable resources and consumes less energy compared to chemical process. Several companies (solely or in partnership) are now scaling bio-succinate production processes which have traditionally suffered from poor productivity and high downstream processing costs. Mitsubishi Chemical (Japan) has developed biomass-derived succinic acid in collaboration with Ajinomoto to commercialize bio-based PBS. DSM and Roquette are developing a commercially feasible fermentation process for the production of succinic acid 1,4-butanediol and subsequent production of PBS. Myriant and Bioamber have developed a fermentation technology to produce monomers. There are several companies around the world developing technologies for the production of PBS, as listed in Table 6, including North America and China (Doug 2010; Ravenstijn 2010).

**Table 6:** **Global producers of PBS**

Company	Location	Brand name/polymer type	Production/planned capacity (kton/year)
BASF	Germany	PBS	
Dupont de Nemours	USA	PBST	
Hexing Chemical	China	PBS	3
Ube	Japan	NA	NA
IPC-CAS	China	PBS, PBSA	5
IRE Chemical	Korea	Enpol, PBS, PBSA	3.5
Kingfa	China	PBSA	1
Mitsubishi Gas Chemical	Japan	PBS, PES, PBSLa	3
Showa	Japan	Bionelle PBS, PBSA, PBS	3
SK Chemicals	Korea	Skygreen	NA
DSM	Netherlands	NA	NA

NA, not available; PBSA, poly(butylene succinate adipate).

Conventional processes for the production of 1,4-butanediol use fossil fuel feedstocks such as acetylene and formaldehyde. The bio-based process involves the use of glucose from renewable resources to produce succinic acid followed by a chemical reduction to produce butanediol. PBS is produced by transesterification, direct polymerization, and condensation polymerization reactions. PBS copolymers can be produced by adding a third monomer such as sebacic acid, adipic acid, and succinic acid which is also produced by renewable resources (Bechthold et al. 2008).

PBS is a semicrystalline polyester with a melting point higher than that of PLA. Its mechanical and thermal properties depend on the crystal structure and the degree of crystallinity (Nicolas et al. 2011). PBS displays similar crystallization behavior and mechanical properties to those of polyolefin such as polyethylene. It has a good tensile and impact strength with moderate rigidity and hardness. The *T* g is approximately −32°C, and the melting temperature is approximately 115°C. In comparison with PLA, PBS is tougher in nature but with a lower rigidity and Young's modulus. By changing the monomer composition, mechanical properties can be tuned to suit the required application (Liu et al. 2009a, b).

PBS and their blends have found commercial applications in agriculture, fishery, forestry, construction, and other industrial fields which are listed in Table 7. For example, PBS has been employed as mulch film, packaging, and flushable hygiene products and also used as a non-migrant plasticizer for polyvinyl chloride (PVC). In addition, it is used in foaming and food packaging application. The relatively poor mechanical flexibility of PBS limits the applications of 100% PBS-based products. However, this can be overcome by blending PBS with PLA or starch to improve the mechanical properties significantly, providing properties similar to that of polyolefin (Eslmai and Kamal 2013; Zhao et al. 2010).

**Table 7:** **Applications of PBS and their blends**

Polymer type	Applications	Reference
PBS/PLA blend	Packaging films, dishware, fibers, medical materials	Weraporn et al. 2011; Liu et al. 2009 a, b; Bhatia et al. 2007; Lee and Wang 2006
PBS and blends	Drug encapsulation systems	Cornelia et al. 2011
PBS/starch	Barrier films	Jian-Bing et al. 2011
PBS and copolymers	Industrial applications	Jun and Bao-Hua 2010 a, b
PBS ionomers	Orthopedic applications	Jung et al. 2009

### Bio-polyethylene

Polyethylene (PE) is an important engineering polymer traditionally produced from fossil resources. PE is produced by polymerization of ethylene under pressure, temperature, in the presence of a catalyst. Traditionally, ethylene is produced through steam cracking of naphtha or heavy oils or ethanol dehydration. With increases in oil prices, microbial PE or green PE is now being manufactured from dehydration of ethanol produced by microbial fermentation. The concept of producing PE from bioethanol is not a particularly new one. In the 1980s, Braskem made bio-PE and bio-PVC from bioethanol. However, low oil prices and the limitations of the biotechnology processes made the technology unattractive at that time (de Guzman 2010).

Currently, bio-PE produced on an industrial scale from bioethanol is derived from sugarcane. Bioethanol is also derived from biorenewable feedstocks, including sugar beet, starch crops such as maize, wood, wheat, corn, and other plant wastes through microbial strain and biological fermentation process. In a typical process, extracted sugarcane juice with high sucrose content is anaerobically fermented to produce ethanol. At the end of the fermentation process, ethanol is distilled in order to remove water and to yield azeotropic mixture of hydrous ethanol. Ethanol is then dehydrated at high temperatures over a solid catalyst to produce ethylene and, subsequently, polyethylene (Guangwen et al. 2007; Luiz et al. 2010).

Bio-based polyethylene has exactly the same chemical, physical, and mechanical properties as petrochemical polyethylene. Braskem (Brazil) is the largest producer of bio-PE with 52% market share, and this is the first certified bio-PE in the world. Similarly, Braskem is developing other bio-based polymers such as bio-polyvinyl chloride, bio-polypropylene, and their copolymers with similar industrial technologies. The current Braskem bio-based PE grades are mainly targeted towards food packing, cosmetics, personal care, automotive parts, and toys. Dow Chemical (USA) in cooperation with Crystalsev is the second largest producer of bio-PE with 12% market share. Solvay (Belgium), another producer of bio-PE, has 10% share in the current market. However, Solvay is a leader in the production of bio-PVC with similar industrial technologies. China Petrochemical Corporation also plans to set up production facilities in China to produce bio-PE from bioethanol (Haung et al. 2008).

Bio-PE can replace all the applications of current fossil-based PE. It is widely used in engineering, agriculture, packaging, and many day-to-day commodity applications because of its low price and good performance. Table 8 shows applications of bio-PE in different fields where it can replace conventional PE.

**Table 8:** **Application of bio-PE polymer and their blends**

Polymer type	Applications	Reference
Bio-PE	Plastics bags, milk and water bottles, food packaging films, toys	Vona et al. 1965; Aamer et al. 2008
Bio-PE and blends	Agricultural mulch films	Kasirajan and Ngouajio 2012

### Bio-based natural polymers

This group consists of naturally occurring polymers such as cellulose, starch, chitin, and various polysaccharides and proteins. These materials and their derivatives offer a wide range of properties and applications. In this section, some of the natural bio-based polymers and their applications in various fields are discussed.

#### Starch

Starch is a unique bio-based polymer because it occurs in nature as discrete granules. Starch is the end product of photosynthesis in plants - a natural carbohydrate-based polymer that is abundantly available in nature from various sources including wheat, rice, corn, and potato. Essentially, starch consists of the linear polysaccharide amylose and the highly branched polysaccharide amylopectin. In particular, thermoplastic starch is of growing interest within the industry. The thermal and mechanical properties of starch can vary greatly and depend upon such factors as the amount of plasticizer present. The *T* g varies between −50°C and 110°C, and the modulus is similar to polyolefins (Jane 1995). Several challenges exist in producing commercially viable starch plastics. Starch's molecular structure is complex and partly nonlinear, leading to issues with ductility. Starch and starch thermoplastics suffer from the phenomenon of retrogradation - a natural increase in crystallinity over time, leading to increased brittleness. Plasticizers need to be found to create starch plastics with mechanical properties comparable to polyolefin-derived packaging. Plasticized starch blends and composites and/or chemical modifications may overcome these issues, creating biodegradable polymers with sufficient mechanical strength, flexibility, and water barrier properties for commercial packaging and consumer products (Maurizio et al. 2005).

Novamont is one of the leading companies in processing starch-based products (Li et al. 2009). The company produces various types of starch-based products using proprietary blend formulations. There are other companies around the world producing starch-based products in a similar scale for various applications, which are listed in Table 9 (Doug 2010; Ravenstijn 2010).

**Table 9:** **Global suppliers of starch-based products**

Company	Location	Brand name	Production/planned capacity (kton/year)
Novamont	Italy	Mater-Bi	120
Japan Corn Starch	Japan	Ever Corn	NA
Biotec	Germany	Bioplast	NA
Rodenberg	Netherlands	Solanyl	50
BIOP	Germany	Biopar	5
Plantic	Australia	Plantic	7.5
Wuhan Huali Environment Protection Sci. & Tech	China	PSM	15
Biograde	China	Cardia	3
PSM	USA	Plaststarch	NA
Livan	Canada	Livan	10

Applications of thermoplastic starch polymers include films, such as for shopping, bread, and fishing bait bags, overwraps, flushable sanitary product, packing materials, and special mulch films. Potential future applications could include foam loose-fill packaging and injection-molded products such as ‘take-away’ food containers. Starch and modified starches have a broad range of applications both in the food and non-food sectors. In Europe in 2002, the total consumption of starch and starch derivatives was approximately 7.9 million tons, of which 54% was used for food applications and 46% in non-food applications (Frost & Sullivan report 2009).

The largest users of starch in the European Union (30%) are the paper, cardboard, and corrugating industries (Frost & Sullivan report 2009). Other important fields of starch application are textiles, cosmetics, pharmaceuticals, construction, and paints, which are listed in Table 10. In the medium and long term, starch will play an increasing role in the field of ‘renewable raw materials’ for the production of biodegradable plastics, packaging material, and molded products.

**Table 10:** **Application of starch and their blends in various fields**

Polymer type	Applications	Reference
Starch	Orthopedic implant devices as bone fillers	Ashammakhi and Rokkanen 1997
Starch/ethylene vinyl alcohol/HA starch/polycaprolactone blends	Bone replacement/fixation implants, orthopedic applications	Mainil et al. 1997; Mendes et al. 2001; Marques and Reis 2005
Starch/cellulose acetate blends with methylmethacrylate and acrylic acid	Bone cements	Espigares et al. 2002
Modified starch	Food applications	Jaspreet et al. 2007; Fuentes et al. 2010
Starch derivatives	Drug delivery	Asha and Martins 2012
Thermoplastic starch	Packaging, containers, mulch films, textile sizing agents, adhesives	Zhao et al. 2008; Maurizio et al. 2005; Ozdemir and Floros 2004; Dave et al. 1999; Guo et al. 2005; Kumbar et al. 2001; Li et al. 2011

#### Cellulose

Cellulose is the predominant constituent in cell walls of all plants. Cellulose is a complex polysaccharide with crystalline morphology. Cellulose differs from starch where glucose units are linked by β-1,4-glycosidic bonds, whereas the bonds in starch are predominantly α-1,4 linkages. The most important raw material sources for the production of cellulosic plastics are cotton fibers and wood. Plant fiber is dissolved in alkali and carbon disulfide to create viscose, which is then reconverted to cellulose in cellophane form following a sulfuric acid and sodium sulfate bath. There are currently two processes used to separate cellulose from the other wood constituents (Yan et al. 2009). These methods, sulfite and pre-hydrolysis kraft pulping, use high pressure and chemicals to separate cellulose from lignin and hemicellulose, attaining greater than 97% cellulose purity. The main derivatives of cellulose for industrial purposes are cellulose acetate, cellulose esters (molding, extrusion, and films), and regenerated cellulose for fibers.

Cellulose is a hard polymer and has a high tensile strength of 62 to 500 MPa and elongation of 4% (Bisanda and Ansell 1992; Eichhorn et al. 2001). In order to overcome the inherent processing problems of cellulose, it is necessary to modify, plasticize, and blend with other polymers. The mechanical and thermal properties vary from blend to blend depending on the composition. The *T* g of cellulosic derivatives ranged between 53°C and 180°C (Picker and Hoag 2002).

Eastman Chemical is a major producer of cellulosic polymers. FKuR launched a biopolymer business in the year 2000 and has a capacity of 2,800 metric ton/year of various cellulosic compounds for different applications (Doug 2010). The major producers of cellulose-based compounds are listed in Table 11 (Doug 2010; Ravenstijn 2010).

**Table 11:** **Global suppliers of cellulosic products**

Company	Location	Brand name
Innovia films	UK	Nature Flex
Eastman Chemical	USA	Tenite
FKuR	Germany	Biograde
Sateri	China	Sateri

There are three main groups of cellulosic polymers that are produced by chemical modification of cellulose for various applications. Cellulose esters, namely cellulose nitrate and cellulose acetate, are mainly developed for film and fiber applications. Cellulose ethers, such as carboxymethyl cellulose and hydroxyethyl cellulose, are widely used in construction, food, personal care, pharmaceuticals, paint, and other pharmaceutical applications (Kamel et al. 2008). Finally, regenerated cellulose is the largest bio-based polymer produced globally for fiber and film applications. Regenerated cellulose fibers are used in textiles, hygienic disposables, and home furnishing fabrics because of its thermal stability and modulus (Kevin et al. 2001).

Chemically pure cellulose can be produced using a certain type of bacteria. Bacterial cellulose is characterized by its purity and high strength. It can be used to produce articles with relatively high strength. Currently, applications for bacterial cellulose outside food and biomedical fields are rather limited because of its high price. The other applications include acoustic diaphragms, mining, paints, oil gas recovery, and adhesives. However, the low yields and high costs of bacterial cellulose represent barriers to large-scale industrial applications (Prashant et al. 2009). Table 12 summarizes the applications of cellulose and their compounds in different fields.

**Table 12:** **Application of cellulose and their compounds in various fields**

Polymer type	Applications	Reference
Cellulose esters	Membranes for separation	Kumano and Fujiwara 2008
Carboxylated methyl cellulose	Drug formulations, as binder for drugs, film-coating agent for drugs, ointment base	Chambin et al. 2004; Obae and Imada 1999; Westermark et al. 1999; Hirosawa et al. 2000
Cellulose acetate fibers	Wound dressings	Orawan et al. 2008; Abdelrahman and Newton 2011
Hydroxyethyl cellulose	Spray for clothes polluted with pollen	Hori et al. 2005
Modified celluloses, cellulose whiskers, microfibrous cellulose	Barrier films, water preservation in food packing	Amit and Ragauskas 2009
Cellulose nanofibers	Textile applications	Zeeshan et al. 2013
Cellulose particles	Chromatographic applications, chiral separations	Levison 1993; Arshady 1991a, b

#### Chitin and chitosan

Chitin and chitosan are the most abundant natural amino polysaccharide and valuable bio-based natural polymers derived from shells of prawns and crabs. Currently, chitin and chitosan are produced commercially by chemical extraction process from crab, shrimp, and prawn wastes (Roberts 1997). The chemical extraction of chitin is quite an aggressive process based on demineralization by acid and deproteination by the action of alkali followed by deacetylated into chitosan (Roberts 1997). Chitin can also be produced by using enzyme hydrolysis or fermentation process, but these processes are not economically feasible on an industrial scale (Win and Stevens 2001). Currently, there are few industrial-scale plants of chitin and chitosan worldwide located in the USA, Canada, Scandinavia, and Asia (Ravi Kumar 2000).

Chitosan displays interesting characteristics including biodegradability, biocompatibility, chemical inertness, high mechanical strength, good film-forming properties, and low cost (Marguerite 2006; Virginia et al. 2011; Liu et al. 2012). Chitosan is being used in a vast array of widely varying products and applications ranging from pharmaceutical and cosmetic products to water treatment and plant protection. For each application, different properties of chitosan are required, which changes with the degree of acetylation and molecular weight. Chitosan is compatible with many biologically active components incorporated in cosmetic product composition (Ravi Kumar 2000). Due to its low toxicity, biocompatibility, and bioactivity, chitosan has become a very attractive material in such diverse applications as biomaterials in medical devices and as a pharmaceutical ingredient (Bae and Moo-Moo 2010; Ramya et al. 2012). Chitosan has application in shampoos, rinses, and permanent hair-coloring agents. Chitosan and its derivatives also have applications in the skin care industry. Chitosan can function as a moisturizer for the skin, and because of its lower costs, it might compete with hyaluronic acid in this application (Bansal et al. 2011; Valerie and Vinod 1998; Hafdani and Sadeghinia 2011).

#### Pullulan

Pullulan is a linear water-soluble polysaccharide mainly consisting of maltotriose units connected by α-1,6 glycosidic units. Pullulan was first reported by Bauer (1938) and is obtained from the fermentation broth of *Aureobasidium pullulans*. Pullulan is produced by a simple fermentation process using a number of feedstocks containing simple sugars (Bernier 1958; Catley 1971; Sena et al. 2006). Pullulan can be chemically modified to produce a polymer that is either less soluble or completely insoluble in water. The unique properties of this polysaccharide are due to its characteristic glycosidic linking. Pullulan is easily chemically modified to reduce the water solubility or to develop pH sensitivity, by introducing functional reactive groups, etc. Due to its high water solubility and low viscosity, pullulan has numerous commercial applications including use as a food additive, a flocculant, a blood plasma substitute, an adhesive, and a film (Zajic and LeDuy 1973; Singh et al. 2008; Cheng et al. 2011). Pullulan can be formed into molding articles which can resemble conventional polymers such as polystyrene in their transparency, strength, and toughness (Leathers 2003).

Pullulan is extensively used in the food industry. It is a slow-digesting macromolecule which is tasteless as well as odorless, hence its application as a low-calorie food additive providing bulk and texture. Pullulan possesses oxygen barrier property and good moisture retention, and also, it inhibits fungal growth. These properties make it an excellent material for food preservation, and it is used extensively in the food industry (Conca and Yang 1993). In recent years, pullulan has also been studied for biomedical applications in various aspects, including targeted drug and gene delivery, tissue engineering, wound healing, and even in diagnostic imaging medium (Rekha and Chrndra 2007). Other emerging markets for pullulan include oral care products (Barkalow et al. 2002) and formulations of capsules for dietary supplements and pharmaceuticals (Leathers 2003), leading to increased demand for this unique biopolymer.

#### Collagen and gelatin

Collagen is the major insoluble fibrous protein in the extracellular matrix and in connective tissue. In fact, it is the single most abundant protein in the animal kingdom. There are at least 27 types of collagens, and the structures all serve the same purpose: to help tissues withstand stretching. The most abundant sources of collagen are pig skin, bovine hide, and pork and cattle bones. However, the industrial use of collagen is obtained from nonmammalian species (Gomez-Guille et al. 2011). Gelatin is obtained through the hydrolysis of collagen. The degree of conversion of collagen into gelatin depends on the pretreatment, function of temperature, pH, and extraction time (Johnston-Banks 1990).

Collagen is one of the most useful biomaterials due to its biocompatibility, biodegradability, and weak antigenicity (Maeda et al. 1999). The main application of collagen films in ophthalmology is as drug delivery systems for slow release of incorporated drugs (Rubin et al. 1973). It was also used for tissue engineering including skin replacement, bone substitutes, and artificial blood vessels and valves (Lee et al. 2001).

The classical food, photographic, cosmetic, and pharmaceutical applications of gelatin is based mainly on its gel-forming properties. Recently in the food industry, an increasing number of new applications have been found for gelatin in products in line with the growing trend to replace synthetic agents with more natural ones (Gomez-Guille et al. 2011). These include emulsifiers, foaming agents, colloid stabilizers, biodegradable film-forming materials, and microencapsulating agents.

#### Alginates

Alginate is a linear polysaccharide that is abundant in nature as it is synthesized by brown seaweeds and by soil bacteria (Draget et al. 1997). Sodium alginate is the most commonly used alginate form in the industry since it is the first by-product of algal purification (Draget 2000). Sodium alginate consists of α-*l*-guluronic acid residues (G blocks) and β-*d*-mannuronic acid residues (M blocks), as well as segments of alternating guluronic and mannuronic acids.

Although alginates are a heterogeneous family of polymers with varying content of G and M blocks depending on the source of extraction, alginates with high G content have far more industrial importance (Siddhesh and Edgar 2012). The acid or alkali treatment processes used to make sodium alginate from brown seaweeds are relatively simple. The difficulties in processing arise mainly from the separation of sodium alginate from slimy residues (Black and Woodward 1954). It is estimated that the annual production of alginates is approximately 38,000 tons worldwide (Helgerud et al. 2009).

Alginates have various industrial uses as viscosifiers, stabilizers, and gel-forming, film-forming, or water-binding agents (Helga and Svein 1998). These applications range from textile printing and manufacturing of ceramics to production of welding rods and water treatment (Teli and Chiplunkar 1986; Qin et al. 2007; Xie et al. 2001). The polymer is soluble in cold water and forms thermostable gels. These properties are utilized in the food industry in products such as custard creams and restructured food. The polymer is also used as a stabilizer and thickener in a variety of beverages, ice creams, emulsions, and sauces (Iain et al. 2009).

Alginates are widely used as a gelling agent in pharmaceutical and food applications. Studies into their positive effects on human health have broadened recently with the recognition that they have a number of potentially beneficial physiological effects in the gastrointestinal tract (Peter et al. 2011; Mandel et al. 2000). Alginate-containing wound dressings are commonly used, especially in making hydrophilic gels over wounds which can produce comfortable, localized hydrophilic environments in healing wounds (Onsoyen 1996). Alginates are used in controlled drug delivery, where the rate of drug release depends on the type and molecular weight of alginates used (Alexnader et al. 2006; Goh et al. 2012). Additionally, dental impressions made with alginates are easy to handle for both dentist and patient as they fast set at room temperature and are cost-effective (Onsoyen 1996). Recent studies show that alginates can be effective in treating obesity, and currently, various functional alginates are being evaluated in human clinical trials (Georg et al. 2012).

### Current status and future trends

The use of bio-based feedstocks in the chemical sector is not a novel concept. They have been industrially feasible on a large scale for more than a decade. However, the price of oil was so cost-effective, and the development of oil-based products created so many opportunities that bio-based products were not prioritized at the time. Several factors, such as the limitations and uncertainty in supplies of fossil fuels, environmental considerations, and technological developments, accelerated the advancement of bio-based polymers and products. It took more than a century to evolve the fossil fuel-based chemical industry; however, the bio-based polymer industry is already catching up with fossil fuel-based chemical industry, which has augmented in the last 20 years. Thanks to advancements in white biotechnology, the production of bio-based polymers and other chemicals from renewable resources has become a reality. The first-generation technologies mainly focused on food resources such as corn, starch, rice, etc. to produce bio-based polymers. As the food-versus-fuel debate ascended, the focus of technologies diverted to cellulose-based feedstocks, focusing on waste from wood and paper, food industries, and even stems and leaves and solid municipal waste streams. More and more of these technologies are already in the pipeline to align with the abovementioned waste streams; however, it may take another 20 years to develop the full spectrum of chemicals based on these technologies (Michael et al. 2011).

Challenges that need to be addressed in the coming years include management of raw materials, performance of bio-based materials, and their cost for production. Economy of scale will be one of the main challenges for production of bio-based monomers and bio-based polymers from renewable sources. Building large-scale plants can be difficult due to the lack of experience in new technologies and estimation of supply/demand balance. In order to make these technologies economically viable, it is very important to develop (1) logistics for biomass feedstocks, (2) new manufacturing routes by replacing existing methods with high yields, (3) new microbial strains/enzymes, and (4) efficient downstream processing methods for recovery of bio-based products.

The current bio-based industry focus is mainly on making bio-versions of existing monomers and polymers. Performance of these products is well known, and it is relatively easy to replace the existing product with similar performance of bio-versions. All the polymers mentioned above often display similar properties of current fossil-based polymers. Recently, many efforts are seen towards introducing new bio-based polymers with higher performance and value. For example, Nature Works LLC has introduced new grades of PLA with higher thermal and mechanical properties. New PLA-tri block copolymers have been reported to behave like thermoplastic elastomer. Many developments are currently underway to develop various polyamides, polyesters, polyhydroxyaloknates, etc. with a high differentiation in their final properties for use in automotive, electronics, and biomedical applications.

The disadvantage of some of the new bio-based polymers is that they cannot be processed in all current processing equipment. There is vast knowledge on additive-based chemistry developed for improving the performance and processing of fossil fuel-based polymers, and this knowledge can be used to develop new additive chemistry to improve the performance and properties of bio-based polymers (Ray and Bousmina 2005). For bio-based polymers like PLA and PHA, additives are being developed to improve their performance, by blending with other polymers or making new copolymers. However, the additive market for bio-based polymers is still very small, which makes it difficult to justify major development efforts according to some key additive supplier companies.

The use of nanoparticles as additives to enhance polymer performance has long been established for petroleum-based polymers. Various nano-reinforcements currently being developed include carbon nanotubes, graphene, nanoclays, 2-D layered materials, and cellulose nanowhiskers. Combining these nanofillers with bio-based polymers could enhance a large number of physical properties, including barrier, flame resistance, thermal stability, solvent uptake, and rate of biodegradability, relative to unmodified polymer resin. These improvements are generally attained at low filler content, and this nano-reinforcement is a very attractive route to generate new functional biomaterials for various applications.

Even though new bio-based polymers are produced on an industrial scale, there are still several factors which need to be determined for the long-term viability of bio-based polymers. It is expected that there will be feedstock competition as global demand for food and energy increases over time. Currently, renewable feedstocks used for manufacturing bio-based monomers and polymers often compete with requirements for food-based products. The expansion of first-generation bio-based fuel production will place unsustainable demands on biomass resources and is as much a threat to the sustainability of biochemical and biopolymer production as it is to food production (Michael et al. 2011). Indeed the European commission has altered its targets downwards for first-generation biofuels since October 2012, indicating its preference for non-food sources of sugar for biofuel production (EurActiv.com 2012). Several initiatives are underway to use cellulose-based feedstocks for the production of usable sugars for biofuels, biochemicals, and biopolymers (Jong et al. 2010).

## Conclusions

Bio-based polymers are closer to the reality of replacing conventional polymers than ever before. Nowadays, bio-based polymers are commonly found in many applications from commodity to hi-tech applications due to advancement in biotechnologies and public awareness. However, despite these advancements, there are still some drawbacks which prevent the wider commercialization of bio-based polymers in many applications. This is mainly due to performance and price when compared with their conventional counterparts, which remains a significant challenge for bio-based polymers.

## Authors’ original submitted files for images

Below are the links to the authors’ original submitted files for images. Authors’ original file for figure 1 Authors’ original file for figure 2
