# Supplementary figures and images for: Current progress on bio-based polymers and their future trends
Source: Prog Biomater. 2013 Mar 18;2:8. doi: 10.1186/2194-0517-2-8 (PMC5151099; doi:10.1186/2194-0517-2-8)

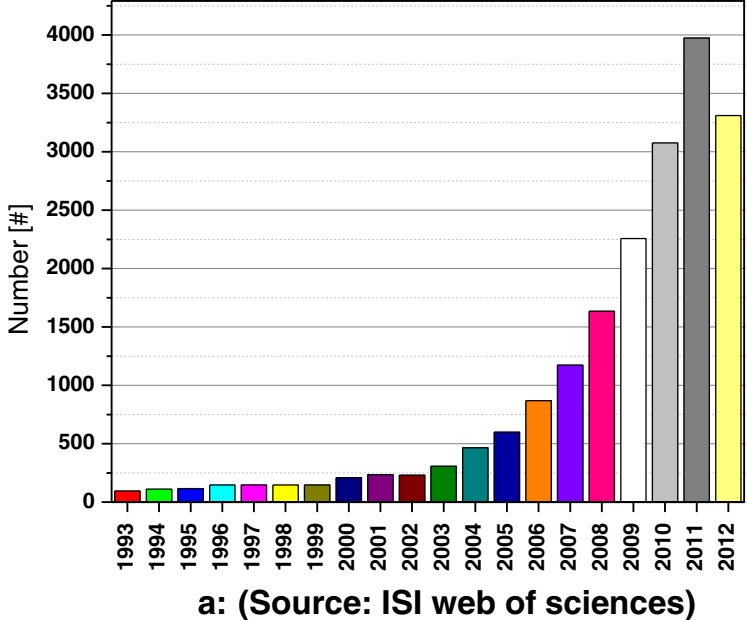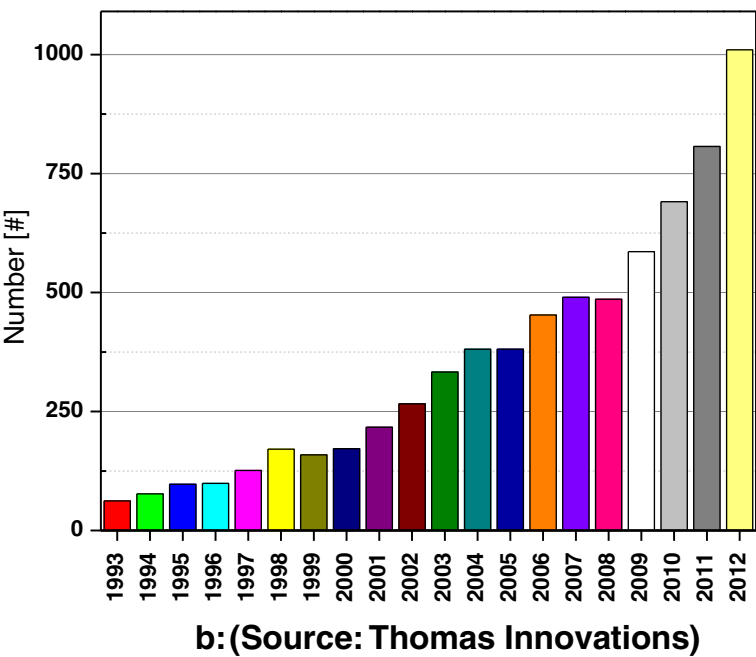

Supplement: Supplementary file 1 — Authors’ original file for figure 1 [file 40204_2012_10_MOESM1_ESM.pdf]
