# Supplementary material for: Current progress on bio-based polymers and their future trends
Source: Prog Biomater. 2013 Mar 18;2:8. doi: 10.1186/2194-0517-2-8 (PMC5151099; doi:10.1186/2194-0517-2-8)
Supplement: Supplementary file 2 — Authors’ original file for figure 2 [file 40204_2012_10_MOESM2_ESM.pdf]

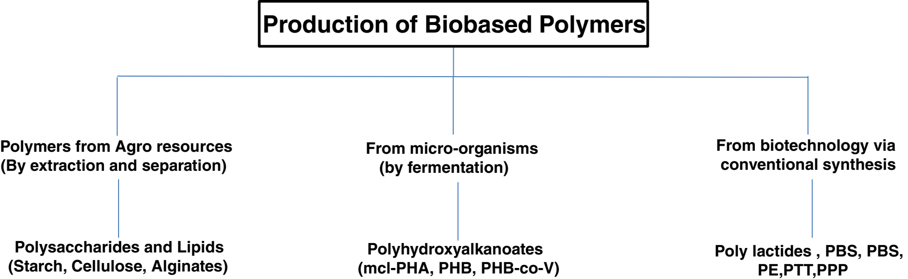
Production of Biobased Polymers
Polymers from Agro resources
(By extraction and separation)
Polysaccharides and Lipids
(Starch, Cellulose, Alginates)
From micro-organisms
(by fermentation)
Polyhydroxyalkanoates
(mcl-PHA, PHB, PHB-co-V)
From biotechnology via
conventional synthesis
Poly lactides , PBS, PBS,
PE,PTT,PPP
